# Accuracy of PlusOptix A09 distance refraction in pediatric myopia and hyperopia

**DOI:** 10.1186/s12886-016-0247-8

**Published:** 2016-06-01

**Authors:** Arnaud Payerols, Claudie Eliaou, Véronique Trezeguet, Max Villain, Vincent Daien

**Affiliations:** Department of Ophthalmology, Gui De Chauliac Hospital, CHU de Montpellier, 80, avenue Augustin Fliche, 34295 cedex 5, Montpellier, F-34000 France; Univ Montpellier 1, Montpellier, F-34000 France; Inserm, U1061, Montpellier, F-34093 France

**Keywords:** Refraction, Cycloplegia, Amblyopia, Screening

## Abstract

**Background:**

The PlusOptix photoscreeners (PlusOptix GmbH, Nuremberg, Germany) is used in many vision screening programs. The purpose of the present study was to further explore the accuracy of the PlusOptix A09 photoscreener in children with ametropia (myopia or hyperopia).

**Methods:**

A total of 70 eyes (35 children) were prospectively included. Before administration with the cycloplegia treatment 1 % cyclopentolate hydrochloride, children underwent refraction measurement with the PlusOptix A09. A refraction was then performed after cycloplegia with either Retinomax hand-held or Nidek autorefractor before and after 3 years old, respectively.

**Results:**

The median (interquartile range) age was 58 (18 to 86) months. The mean (SD) spherical equivalent differed between PlusOptix A09 and cycloplegic autorefraction (+0.54 [1.82] D vs +1.06 [2.04] D, *p* = 0.04). PlusOptix A09 refraction was positively correlated with cycloplegic autorefraction (*r* = 0.81, *p* < 0.001) with higher coefficient in myopic than in hyperopic children (*r* = 0.91, *p* = 0.0002 and *r* = 0.52, *p* = 0.01, respectively). The mean (SD) difference between PlusOptix A09 and cycloplegic autorefraction was higher with hyperopia than myopia (0.73 [1.34] vs 0.05 [0.66], *p* = 0.01). The proportion of children with < 1-D difference between cycloplegic and PlusOptix A09 refraction was 68.8 %, higher with myopia than hyperopia (90 % vs 54.5 %, *p* = 0.01).

**Conclusion:**

The spherical equivalent value with non-cycloplegic PlusOptix A09 refraction is closer to that with cycloplegic autorefraction than non-cycloplegic autorefraction. The PlusOptix A09 photoscreener underestimated the hyperopia of 0.73 D and slightly overestimated myopia of 0.05 D. The PlusOptix A09 could be used for screening with higher accuracy in myopic than hyperopic children.

## Background

Whole-population screening of children younger than 5 years is applied in many countries. However, the procedures and details of vision screening are inconsistent worldwide. Some studies tended to use photorefraction for early detection of amblyopia risk factors in children’s vision screening. This screening approach was based on the evidence that noncycloplegic photorefraction had acceptable accuracy and advantages of speed and portability when compared with cycloplegic retinoscopy [1]. On the contrary, others regarded photorefraction without cycloplegia as unreliable because of poor accuracy and limited range of refractive errors [2].

A series of the PlusOptix photoscreeners (PlusOptix GmbH, Nuremberg, Germany) are newly designed photorefraction tools for vision screening in children are approved by the US Food and Drug Administration (FDA) as a refractor. There are some reports of the sensitivity and specificity of the PlusOptix S04 or S08 for detecting amblyopia risk factors [3–9]. The PlusOptix A08 had higher predictive values for refractive error than for strabismus screening, with sensitivity 88 and 52 %, respectively [3]. In a study of children 6–36 months old, the PlusOptix S08 photoscreener had high sensitivity (100 %) but low specificity (38.7 %) for detecting amblyopia risk factors as compared with retinoscopy [4]. In a study of 64 patients 2–19 years old, 70.2 % showed a significant difference between PlusOptix S04 without cycloplegia and cycloplegic retinoscopy of > 0.5 D [7].

The purpose of the present study was to further explore the accuracy of the PlusOptix A09 photoscreener in pediatric patients with ametropia (myopia or hyperopia).

## Methods

The present study was approved by the medical ethics committee of Montpellier Hospital and was in accordance with the 1975 Declaration of Helsinki. We performed cycloplegic autorefraction only in patients with this indication. This is in accordance with article R. 1121–3 of French public health law (April 26, 2006). Written, informed consent was provided by the parents of the pediatric patients.

### Study population

We prospectively included consecutive children 12 to 139 months (interquartile range [25–75 %] 18 to 86 months) examined in the ophthalmology department of Gui de Chauliac Hospital of Montpellier. Inclusion criteria were consulting for screening of refractive error or monitoring of strabismus or amblyopia. Children with organic amblyopia, history of congenital cataract, significant media opacities or retinopathy were excluded.

### Refraction assessment

Three different autorefractors were used: autorefractors (Retinomax hand-held and Nidek ARK-530A) and the PlusOptix A09 distance photoscreener.

The portable Retinomax autorefractor was used with its front support for stabilizing the measurement and a vertical mark to determine the correct vertical position. The value of the sphere can be measured from – 18 to + 23 D in increments of 0.25D, and from 0 to + 12 D for the cylinder in increments of 0.25D.

The fixed Nidek ARK-530A autorefractor provides a measure of refraction every 0.3 s. The value of the sphere can be measured from – 30 to + 25 D in increments of 0.01 D, and from 0 to 12 D for the cylinder in increments of 0.01D. We considered the refraction valuable if at least 3 readings were obtained with Retinomax and Nidek ARK-530A with quality control value > 7. In the present analysis, we used the mean value provided by the autorefractor.

The PlusOptix A09 photoscreener was placed at a distance of one meter in front of the patient in a darkroom and operated by a trained nurse. The fixation target of the instrument was designed as a smile face on the camera. Once pressing the start button, the smile face was automatically lighted and a warble sound could be heard to draw the child’s attention to the camera. The children were asked to gaze at the nose of the smile face on the camera during the test. Then the camera was moved slightly (within 50 mm) until green circles were evident around both pupils on the monitor screen, which was followed by automatic measurement. The results were displayed on the monitor. The PlusOptix A09 photoscreener has a spherical and cylindrical range of −7.0 to +5.0 D in increments of 0.25 D. If the spherical equivalent (SE) is out of the range, the measurement value only displays “Hyperopia” or “Myopia”. Ocular misalignment ≥10° could not be measured binocularly, and was changed to a sequential monocular measurement mode. Each patient was tested twice and the average value was the final result.

### Study protocol

Before cycloplegia treatment, all children underwent refraction measurement with the PlusOptix A09 and an autorefractor depending on the age of the patient (Retinomax hand-held before 3 years and Nidek autorefractor after 3 years). Measurements with a autorefractor were also performed after administration of 1 % cyclopentolate hydrochloride.

### Cycloplegia

Cycloplegia treatment with 1 % cyclopentolate hydrochloride was used according to the following protocol: 1 drop every 0, 5 and 10 min and refraction measured 45 to 60 min after the first drop.

### Statistical analysis

We first compared the mean (SD), medians and the interquartile ranges of spherical equivalence between the right and left eyes. Myopia and hyperopia gold standards were defined by cycloplegic autorefraction. Paired *t* tests were used to compare spherical equivalents, sphere, cylinder, axis and anisometropia before and after cycloplogia. The Bonferroni correction was used to correct *p*-values of the main comparison of spherical equivalent between non-cycloplegic photorefraction PlusOptix and cycloplegic refraction. Pearson coefficients were used to correlate refraction values. The Bland-Altman method was used to assess the difference in refraction with the PlusOptix A09 and cycloplegic autorefraction. The mean difference and difference in means between PlusOptix A09 and cycloplegic autorefraction were plotted. SAS 9.4 (SAS Inst., Cary, NC) was used for data analysis. Significance was set at *P* < 0.05.

## Results

### Population characteristics

We initially examined 35 children; 3 were excluded from the analysis because the PlusOptix A09 measurement failed (hyperopia = 10 D for *n* = 1 and excessive mydriasis for *n* = 2). All patients underwent non-cycloplegic and cycloplegic autorefraction. The 32 children included 16 boys; the median (interquartile range) age was 58 months (18–86) months (11 children < 3 years old and 21 children > 3 years old). Most children consulted for follow-up for refractive error (75 %) or for monitoring amblyopia (15.6 %) or strabismus (9.4 %). After cycloplegia, 14 children were myopic and 18 hyperopic.

Values for right and left eyes did not differ for spherical equivalents with the non-cycloplegic autorefraction, PlusOptix A09 and cycloplegic autorefraction (*p* = 0.16, *p* = 0.55 and *p* = 0.21, respectively). Therefore, the right eye was used for all analyses.

### Comparison of spherical equivalents between non-cycloplegic autorefraction, PlusOptix A09 and cycloplegic autorefraction

Mean (SD) spherical equivalents differed between non-cycloplegic autorefraction and the PlusOptix A09 (−0.70 [3.14] vs 0.54 [1.82] D, *p* = 0.02), between spherical equivalents with the PlusOptix A09 and cycloplegic autorefraction (0.54 [1.82] vs 1.06 [2.04] D, *p* = 0.04) and between spherical equivalents with the non-cycloplegic autorefraction and cycloplegic autorefraction (−0.70 [3.14] vs 1.06 [2.04] D, *p* = 0.004, Table 1).

**Table 1:** Spherical equivalents, sphere, cylinder and axis for each method for the right eye

	Spherical equivalent	Sphere	Cylinder	Axis
Non-cycloplegic autorefraction (*N* = 32)	−0.70 (3.14)^a^	−0.02 (3.33)^d^	−1.16 (1.35)^g^	72.75 (66.00)^j^
Nidek (*N* = 21)	0.04 (2.57)	0.76 (2.72)	−1.13 (1.51)	82.19 (72.05)
Retinomax (*N* = 11)	−2.10 (3.74)	−1.50 (3.98)	−1.21 (1.05)	54.73 (50.79)
PlusOptix A09	0.54 (1.83)^b^	1.27 (2.07)^e^	−1.46 (1.22)^h^	84.41 (68.56)^k^
Cycloplegic autorefraction (*N* = 32)	1.06 (2.04)^c^	1.77 (2.20)^f^	−1.41 (0.98)^i^	73.50 (67.29)^l^
Nidek (*N* = 21)	1.06 (2.29)	1.75 (2.46)	−1.38 (1.09)	75.14 (71.47)
Retinomax (*N* = 11)	1.06 (1.57)	1.80 (1.71)	−1.48 (0.79)	70.36 (61.69)

Data are mean (SD)

^a^ *P* = 0.004 between autorefractor (Nidek/Retinomax) and cycloplegic autorefraction

^b^ *P* = 0.02 between autorefractor (Nidek/Retinomax) and PlusOptix A09

^c^ *P* = 0.04 between PlusOptix A09 and cycloplegic autorefraction

^d^ *P* < 0.01 between autorefractor (Nidek/Retinomax) and cycloplegic autorefraction

^e^ *P* = 0.007 between autorefractor (Nidek/Retinomax) and PlusOptix A09

^f^ *P* = 0.044 between PlusOptix A09 and cycloplegic autorefraction

^g^ *P* = 0.09 between autorefractor (Nidek/Retinomax) and cycloplegic autorefraction

^h^ *P* = 0.19 between autorefractor (Nidek/Retinomax) and PlusOptix A09

^i^ *P* = 0.69 between PlusOptix A09 and cycloplegic autorefraction

^j^ *P* < 0.01 between autorefractor (Nidek/Retinomax) and cycloplegic autorefraction

^k^ *P* = 0.16 between autorefractor (Nidek/Retinomax) and PlusOptix A09

^l^ *P* = 0.28 between PlusOptix A09 and cycloplegic autorefraction

Correlation of refraction value between

-PlusOptix A09 and cycloplegic autorefraction: *r* = 0.81, *p* < 0.001

-PlusOptix A09 and non-cycloplegic autorefraction: *r* = 0.70, *p* < 0.001

-Non-cycloplegic and cycloplegic autorefraction: *r* = 0.77, *p* < 0.001

### Comparison of sphere, cylinder and axis among non-cycloplegic autorefraction, PlusOptix A09 and cycloplegic autorefraction

As shown in Table 1, we observed significant differences in mean sphere, cylinder and axis values between non-cycloplegic autorefraction (−0.02 D, −1.16 D and 72.75°, respectively), PlusOptix A09 (+1.27 D, −1.46 D and 84.41°, respectively) and cycloplegic autorefraction (+1.77 D, −1.41 D and 73.50°, respectively). Sphere values were significant between non-cycloplegic autorefraction and PlusOptix A09 (*p* = 0.007) and between PlusOptix A09 and cycloplegic autorefraction (*p* = 0.044). All comparisons concerning cylinder and axes were not statistically significant among non-cycloplegic, PlusOptix and cycloplegic autorefraction.

### Anisometropia observed with each refraction method

The frequency of anisometropia > 1 D for PlusOptix A09, non-cycloplegic and cycloplegic autorefraction was 15.63, 37.50 and 12.50 %, respectively. Anisometropia differed between PlusOptix A09 and cycloplegic autorefraction (*p* = 0.04) and between non-cycloplegic and cycloplegic autorefraction (*p* = 0.005).

### Comparison of difference (PlusOptix A09 – cycloplegic autorefraction) between myopic and hyperopic children

The mean (SD) difference in spherical equivalents between PlusOptix A09 and cycloplegic autorefraction was higher for hyperopic than myopic children (0.73 [1.34] vs 0.05 [0.66], *p* = 0.01) (Table 2). The proportion of children with < 1-D difference between cycloplegic and PlusOptix A09 refraction was 68.8 %, higher with myopia than hyperopia (90 % vs 54.5 %, *p* = 0.01).

**Table 2:** Mean difference in spherical equivalents between PlusOptix A09 and cycloplegic autorefraction among children with hyperopia and myopia measured by cycloplegia

	Total	Hyperopia (*n* = 18)	Myopia (*n* = 14)	*P* value
Difference between PlusOptix A09 and cycloplegia autorefraction mean (SD)	0.52 (1.2)	0.73 (1.34)	0.05 (0.66)	0.01
Children with < 1-D difference between PlusOptix A09 and cycloplegia autorefraction (%)	68.75 %	54.5 %	90.0 %	0.01

Correlation between cycloplegic autorefraction with PlusOptix A09 and cycloplegic autorefraction

-in myopic children (*r* = 0.91, *p* = 0.0002)

-in hyperopic children (*r* = 0.52, *p* = 0.01)

### Correlation between PlusOptix A09 and autorefraction

**Fig. 1:**
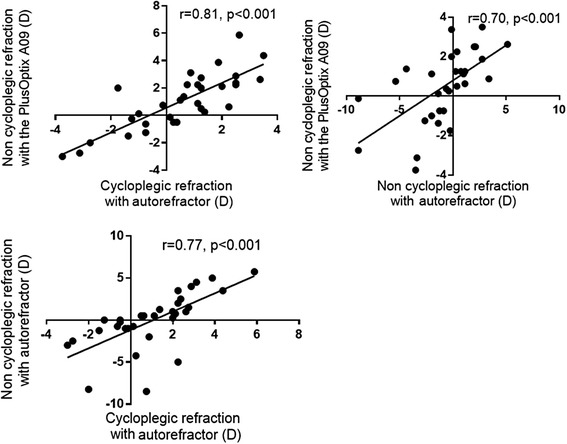
Correlation between autorefractor and PlusOptix A09. *Top left graph*: Correlation between non-cycloplegic autorefraction with PlusOptix A09 and cycloplegic autorefraction with a autorefractor (*r* = 0.81, *p* < 0.001); *top right graph*: non-cycloplegic autorefraction with PlusOptix A09 and with autorefractor (*r* = 0.70, *p* < 0.001), *down left graph* non-cycloplegic autorefraction and cycloplegic autorefraction (*r* = 0.77, *p* < 0.001)

Figure 1 shows the correlation between non-cycloplegic photorefraction with PlusOptix A09 and cycloplegic autorefraction (*r* = 0.81, *p* < 0.001); the correlation between non-cycloplegic photorefraction with PlusOptix A09 and non-cycloplegic autorefraction (*r* = 0.70, *p* < 0.001); and the correlation between non-cycloplegic autorefraction and cycloplegic autorefraction (*r* = 0.77, *p* < 0.001).

### Bland-Altman analysis

#### Between PlusOptix A09 and cycloplegic autorefraction

**Fig. 2:**
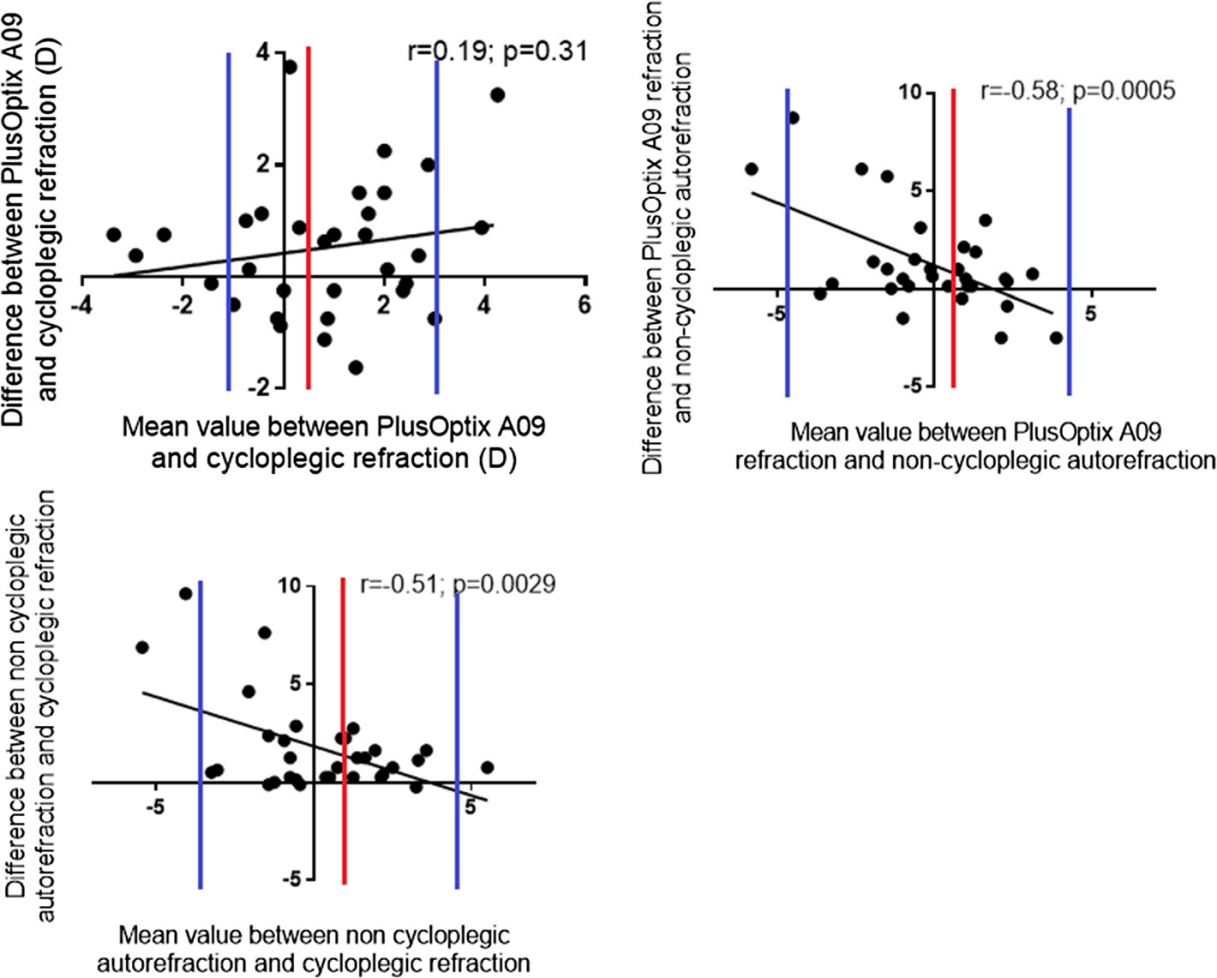
Bland and Altman analysist. *Top left graph*: Correlation between the mean difference and difference in means between non-cycloplegic autorefraction with PlusOptix A09 and cycloplegic autorefraction (*r* = 0.19; *p* = 0.31), red curve: bias (mean difference between the measures = 0.52, blue curves: limit of agreements [−1.55 to +3.15 D]; *top right graph*: Correlation between the mean difference and difference in means between PlusOptix A09 refraction and non-cycloplegic autorefraction (*r* = −0.58; *p* = 0.0005), red curve: biais (mean difference between the measures = 1.24, blue curves: limit of agreements [−4.94 to 4.79 D]; *down left graph*: Correlation between the mean difference and difference in means between non-cycloplegic autorefraction and cycloplegic autorefraction (*r* = −0.51; *p* = 0.0029), red curve: bias (mean difference between the measures = 1.75, blue curves: limit of agreements [−4.42 to 4.79 D]

The mean (SD) difference between the PlusOptix A09 and cycloplegic autorefraction was 0.52 [1.82] D. The mean difference and difference in means did not differ between PlusOptix A09 and cycloplegic autorefraction (*r* = 0.19; *p* = 0.31) (Fig. 2 top left graph), so the difference (cycloplegic autorefraction minus PlusOptixA09) did not increase in extreme values. The 95 % limits of agreement according to the Bland-Altman definition was from −1.55D to +3.15D.

#### Between PlusOptix A09 refraction and non-cycloplegic autorefraction

The mean (SD) difference between the PlusOptix A09 and non-cycloplegic autorefraction was 1.24 (2.48) D. The mean difference and difference in means differed between PlusOptix A09 and non-cycloplegic autorefraction (*r* = −0.58; *p* = 0.0005) (Fig. 2 top right graph), so the difference (non-cycloplegic autorefraction minus PlusOptixA09) was increased in extreme values. The 95 % limits of agreement according to the Bland-Altman definition was from −4.94 to 4.79 D, but they are not statistically valuable because of a significant r correlation coefficient.

#### Between non-cycloplegic autorefraction and cycloplegic autorefraction

The mean (SD) difference between non-cycloplegic and cycloplegic autorefraction was 1.75 (1.82) D. The mean difference and difference in means differed between non-cycoplegic and cycloplegic autorefraction (*r* = −0.51; *p* = 0.0029) (Fig. 2 down left graph), so the difference (non-cycloplegic autorefraction minus cycloplegic autorefraction) was increased in extreme values. The 95 % limits of agreement according to the Bland-Altman definition was from −4.42 to 4.79D, but they are not statistically valuable because of a significant r correlation coefficient.

### Correlation between PlusOptix A09 and cycloplegic autorefraction in myopic and hyperopic children

**Fig. 3:**
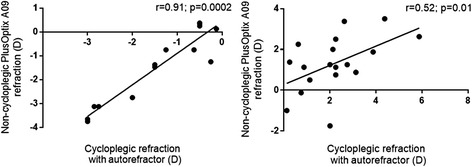
Correlation between non-cycloplegic autorefraction with PlusOptix A09 and cycloplegic autorefraction among myopic and hyperopic children: *left graph*: in myopic children (*r* = 0.91, *p* = 0.0002), *right graph*: in hyperopic children (*r* = 0.52, *p* = 0.01)

Overall the PlusOptix A09 refraction was positively correlated with cycloplegic autorefraction with higher coefficient in myopic than in hyperopic children (*r* = 0.91, *p* = 0.0002 and *r* = 0.52, *p* = 0.01, respectively) (Fig. 3).

## Discussion

The present study provides additional information regarding the performance of the PlusOptix A09 photoscreener in children. The accuracy of the PlusOptix A09 is higher in myopic than hyperopic children and the proportion of children with < 1-D difference between cycloplegic autorefraction and PlusOptix A09 was higher for myopic than hyperopic children.

Studies of diagnostic performance of PlusOptix have found sensitivity 98 to 99 %, specificity 82 to 88 % [10, 11]. With its high sensitivity and acceptable specificity, the PlusOptix A09 can be used in a general screening programs. In myopia screening programs among children ≥ 4 years old, long-distance visual acuity could be used instead of refraction measurement. The benefit of using PlusOptix A09 is to obtain an estimation of severity of myopia [12]. Schimitzek and Lagrèze showed that the mean difference in values between the cycloplegic autorefraction and the photorefractor was 0.73; the spherical equivalent tended to be underestimated because of uncontrolled accommodation in children [13]. In the present study, the non-cycloplegic photorefraction with the PlusOptix A09 was closer to cycloplegic autorefraction than with autorefractors. Non-cycloplegic refraction has lower agreement than use of the PlusOptix A09 because we found a significant correlation between the mean difference and the difference in means between non-cycloplegic and cycloplegic autorefraction. Thus there are more “errors” in extreme values for non-cycloplegic than PlusOptix A09 as compared with cycloplegic autorefraction. We also found an underestimation of the spherical equivalents measured with the PlusOptix A09 as compared with cycloplegic autorefraction but with a smaller difference than in the study performed by Schimitzek et al. (0.52 vs 0.73, respectively). Our result differed from Schimitzek et al. probably because of the younger age of our patients as compared with those in the Schimitzek et al. study (58 months vs. 43 years).

In the present study, the PlusOptix A09 is revealed to have a general trend towards myopic values which is in accordance with previous reports of the PlusOptix photoscreeners [5, 14]. Nevertheless, the diopters shifting to myopia were variable in different reports. Moghaddam et al. [4], reported that the mean difference of SE measured between the PlusOptix and via retinoscopy was 0.16 D on children aged 6 months to 3 years. Erdurmus’s study [15] included a cohort of healthy children (age: 7.1 ± 2.4 years (mean ± SD); range, 9 months to 14 years) and showed that the difference of SE between the PlusOptix and cycloplegic retinoscopy was 0.70 D. Dahlmann-Noor et al. [16], recruited 126 children with a mean age of 5.5 years attending hospital-based pediatric eye service to their study, and confirmed a myopic shift of 1.90 D. The working distance of the PlusOptix A09 is 1.0 m, so stimulation to accommodate does not occur in patients with myopia ≥ −1.0 D because the far point is ≤ 1.0 m. For patients with myopia ≤ −1.0 D, those with emmetropia or hyperopia may accommodate exactly onto the target, and the device will detect a SE = −1.0 D regardless of the real ametropia. Thus, in the present study, a separated analysis was performed between myopic and hyperopic children. The PlusOptix A09 photoscreener underestimated the hyperopia of 0.73 D and slightly overestimated myopia of 0.05 D.

Rajavi et al*.* [17], and Erdurmus et al. [15], studied the relationship between the PlusOptix photoscreener and cycoplegic retinoscopy by means of Pearson correlation. The accuracy of the PlusOptix photoscreener was controversial because the refractive result of the PlusOptix was not consistent with that of cycloplegic retinoscopy [16, 18]. In the present study, we found that linear regression had significant correlation between the PlusOptix photoscreener and cycoplegic refraction with higher coefficient in myopic than in hyperopic children.

Comparison between Retinomax and Nidek was not the aim of this study. Of note, a previous study found that non-cycloplegic Retinomax values were significantly lower than 0.80 D as compared with Nidek values [2].

The limitations of the present study include the low number of children. The paucity of high hyperopes in our study limits the generalization of our conclusions in this high risk of amblyopia population. Also since the subjects involved were patients attending our eye department, the results may be affected by a higher prevalence of eye diseases than those in a healthy population. A population based large-scale photorefraction in a normal child population is underway to further substantiate the results obtained herein. However, the new information comparing myopic and hyperopic children is of interest for users of PlusOptix A09.

## Conclusion

We found that PlusOptix A09 gives closer values for cycloplegic autorefraction than non-cycloplegic autorefraction. The accuracy of the PlusOptix A09 is higher in myopic than hyperopic children. Distance refraction can constitute a tool for screening or follow up that have higher values than non-cycloplegic autorefraction Retinomax/Nidek. However it cannot replace cycloplegic autorefraction for first-spectacle correction and during strabismus or amblyopia management.
